# The Healing of Arteries

**Published:** 1879-08

**Authors:** 


The Healing of Arteries.—One of the most difficult, and, at the same time, important problems in the study of repair is the solution of the mystery enshrouding those processes by which blood-vessels are restored after injury. We have here a combination of conditions essentially different from those which concern the repair of any other structure of the body; for not only the solid vessel, but also its fluid contents, seem to play a conspicuous part in the regenerative changes. It is a subject which has interested surgeons ever since the days of Ambrose Pare, but during the last century and a half it may fairly be said to have claimed the attention of scientific observers. It is a gratifying sign of the tendency of American physicians to devote more of their time to the scientific departments of medicine, to add to, as well as to utilize, medical knowledge, that the recent investigations of Dr. E. O. Shakespeare, of Philadelphia, have enabled us to obtain a much more satisfactory understanding of the nature of the healing process in arteries, and to unravel many of its seeming complications. These were embodied in a paper to which the Warren Triennial Prize for 1877 was awarded, and with a few additions they have recently appeared for the first time in print as No. VII. of the Toner Lectures, published by the Smithsonian Institution.

The question on which observers have been chiefly at variance relates to the part played by the thrombus in the process of repair. Does the thrombus become organized and form the cicatrix, or does it play a perfectly passive role and finally crumble up and disappear, the wall of the vessel doing the work? One view or the other has alternately prevailed, until the discovery of the peculiar habits of the white corpuscles by Cohnheim seemed to throw the balance in favor of the thrombus as a healing agent. Such, in fact, is the position assigned to it by the standard works on pathology to-day, as, for instanoe, Billroth. The views thus freely disseminated have received recent application to the healing of wounds when extensive blood clots under the protecting influence of the Lister dressing are supposed to form the future cicatrical tissue. Moreover, now that the fixed connective tissue cell was considered to take no active part in inflammation, the vessel walls had to be looked upon more than ever as dependent upon the thrombus for the supply of the new tissue. We have not the space to give even a concise summary of the careful and extensive series of experiments of the author.

Suffice it to say that they settle most conclusively that the blood clot has nothing whatever to do with the formation of the cicatrix. It acts simply as a temporary barrier to the course of the blood, and as a foreign body whose tendency is to produce a certain amount of irritation in the adjacent internal coat of the vessel. The healing of an artery after ligature is effected by the formation of a plug of tissue which emanates from the endothelium and subjacent cellular elements of the tunica intima. To this structure he has given the name *plastic clot*, to distinguish it from the *fibrinous or blood clot.* The former develops beneath the latter, and gradually pushes it upward, until, separated from the adjacent walls, it finally disappears. The structure of the blood clot is peculiar, not being a simple, homogeneous mass, as is usually supposed: it has a stratified arrangement lying in spiral coils, the blood column retaining its cylindrical form as it is coiled up in the distended cul-de-sac of the stump. These are a few of the points settled by his investigations, but the tracing of the plastic clot through its various stages of development is alone of great importance as disposing of the organization of the thrombus theory more effectually than any observations with which we are acquainted. Dr. Shakespeare shows the qualities of a good observer, and if he continues his researches in the domain of pathological histology will be sure to give a good account of himself. We would also call attention to the value of such encouragement as the funds of the Warren prize and Toner lectures hold out for good original work of this kind.—*Boston Medical and Surgical Journal.*

